# Exploring genotype–phenotype correlations in pathological myopia: a case report

**DOI:** 10.3389/fmed.2025.1624093

**Published:** 2025-08-05

**Authors:** Qiaoqiao Kong, Xuejing Lu

**Affiliations:** Eye School of Chengdu University of Traditional Chinese Medicine, Chengdu, China

**Keywords:** pathologic myopia, high myopia, posterior scleral staphyloma, RHO gene, gonadal chimerism

## Abstract

**Background:**

Genome-wide association studies have identified key roles for specific genes in ocular axis elongation and related complications in pathological myopia (PM). In this study, we conducted a comprehensive genetic analysis of a family with a high prevalence of PM to identify novel genetic loci associated with PM, aiming to inform clinical practice.

**Materials and methods:**

Genomic DNA was extracted from oral swabs of the proband and family members for sequencing.

**Results:**

A RHO gene variant (NM_000539.3:exon1:c.61C > T:p.R21C) was identified in the proband, potentially associated with the clinical phenotype. Her eldest sister carried the wild-type allele, while her second sister was heterozygous at the validation locus. Further investigation revealed a clustering of female patients with high myopia among the patient’s maternal siblings and their offspring. Therefore, we extended our study to include maternal relatives with axial lengths greater than 26 mm and highly myopic features to identify potential genetic loci. However, exome high-throughput sequencing did not detect any pathogenic variants. Given that the proband’s mother was deceased, whole-exome sequencing was performed on her father and her second sister, who had more severe conditions. No variants were found that could explain the observed clinical phenotype. Thus, we hypothesized that the proband’s mother might carry a gonadal chimeric variant.

**Conclusion:**

The clinical significance of the RHO gene variant (NM_000539.3:exon1:c.61C > T:p.R21C) in our family remains unclear, and the variant is classified as a variant of uncertain significance. Although this RHO variant may potentially be associated with the observed phenotype, further evidence is required to establish a definitive correlation. Based on the available data, gonadal mosaicism represents the most plausible explanatory model; however, this hypothesis cannot be considered conclusive at this stage.

## 1 Introduction

Pathologic myopia (PM) is a severe ocular condition affecting approximately 3% of the global population, with prevalence rates ranging from 0.2 to 1.5% in Asian populations and 0.1 to 0.5% in Caucasian populations, often leading to visual impairment or blindness (1). PM is a leading cause of irreversible blindness in China, particularly among individuals aged 40–49 years. PM not only leads to a loss of best-corrected visual acuity (BCVA) but can also result in blindness, significantly impacting the quality of life and socio-economic status of affected individuals. The pathogenesis of PM is multifactorial, involving the interplay of both environmental and genetic factors. Regarding genetic factors, over 150 genes and 25 genomic loci have been identified as associated with myopia, with nearly 20 loci specifically linked to high myopia (HM) (2). Genome-wide association studies (GWAS) have identified key genes that play a central role in the pathogenesis of abnormal axial elongation and HM. Recent GWAS have identified a significant association between the LILRB2 gene and PM in East Asian populations. These studies have also elucidated how LILRB2 influences lipid metabolism, thereby promoting the development of PM, as demonstrated through animal experiments (3). This discovery not only enhances our understanding of the genetic underpinnings of PM but also identifies novel therapeutic targets for future treatment strategies. PM is characterized by excessive axial elongation, leading to posterior scleral staphyloma, myopic macular degeneration (MMD), and high myopia-associated optic neuropathy (HM-AON). These complications significantly impair visual function, with MMD being a leading cause of vision loss. Several studies have identified candidate genes associated with PM, with CCDC102B emerging as a susceptibility gene for MMD (4). Furthermore, alterations in collagen and growth factor-related genes have been associated with an increased risk of retinal detachment, retinal tears, and retinal holes, as well as the development of myopic choroidal neovascularization. Multiple gene-phenotype association studies have consistently demonstrated that COL2A1 and COL11A1 are strongly associated with PM and its associated fundus lesions in large cohorts, particularly COL2A1 (5–7). Other collagen-related genes associated with intra- and interlayer retinal separation in the context of HM include LEPREL1, matrix metalloproteinases (MMPs), ADAMTS family members, and lumican (8–11). The growth factor-related gene TGF-*β* serves as a common terminal pathway in the development of fundus lesions associated with HM, and its genetic diversity is closely associated with the incidence of HM (12). While numerous studies have uncovered the genetic basis of PM, the intricate genetic mechanisms underlying this condition remain to be fully elucidated. Significant differences in these genes across different populations, as well as the specific mechanisms by which they influence PM, require further investigation. Moreover, the potential pathogenic genes associated with complications of PM exhibit both commonalities and specificities, adding to the complexity of research efforts. The aim of this study is to identify novel gene loci associated with PM through genetic analysis of a familial case, thereby providing a foundation for future research and clinical applications. Given the association between posterior scleral staphyloma and MMD, future investigation of genetic markers linked to these lesions or morphological changes in the posterior pole of the eye will be crucial for elucidating the pathogenesis of PM and developing novel therapeutic strategies.

## 2 Materials and methods

### 2.1 Subjects and clinical evaluations

**Figure 1:**
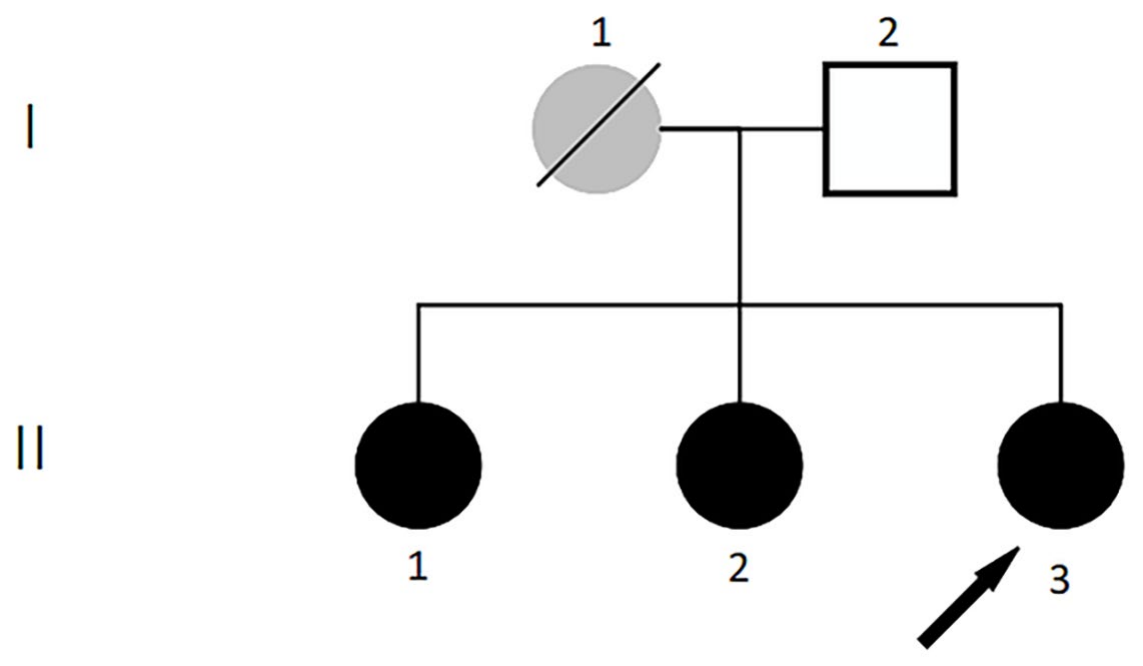
Pedigree of the family with pathological myopia. ●: females affected by pathologic myopia; □: males not affected; 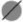: deceased females with high myopia; 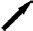: the proband.

On July 12, 2023, the patient underwent posterior scleral reinforcement surgery at the Yinhai Eye Hospital, Chengdu University of Traditional Chinese Medicine, for bilateral PM and posterior scleral staphyloma. She also has hypothyroidism and bilateral cataracts (C1N2). Family history revealed that the proband’s mother and two sisters had HM, with the mother and second sister also diagnosed with hypothyroidism. The second sister underwent posterior scleral reinforcement surgery at our hospital on May 4, 2023, and was diagnosed with: 1. Bilateral pathological myopia; 2. Bilateral scleral staphyloma; 3. Left macular hole with retinal detachment; 4. Left macular split; 5. Left macular membrane; 6. Bilateral concurrent cataract (C1N1). The eldest sister has: 1. Bilateral pathological myopia; 2. Posterior scleral staphyloma in both eyes; 3. Right macular split. She underwent surgery on October 25, 2023. Their father had: 1. Bilateral metabolic cataracts; 2. Type 2 diabetes mellitus; 3. Hyperthyroidism; 4. Hypertension. Based on this information, a family pedigree (Figure 1) was constructed.

PM is characterized by an axial length (AL) exceeding 26 mm and/or a spherical equivalent refraction of ≤ − 6.00 diopters (D), accompanied by atrophic changes in the fundus, macular schisis, or posterior scleral staphyloma. The data for uncorrected visual acuity (UCVA), intraocular pressure (IOP), AL, spherical equivalent refraction, and BCVA of the affected family members are summarized in Table 1.

**Table 1:** Ophthalmic examination parameters of family members shown in Figure 1.

Member ID	Gender	Age(y)	UCVA		IOP (mmHg)		AL (mm)		Refraction & BCVA	
			OD	OS	OD	OS	OD	OS	OD	OS
Ι1	Female	/	/	/	/	/	/	/	/	/
Ι2	Male	79	0.4	0.6	13	13	23.05	23.08	Ph → No improvement	+0.50/−0.50*90 → 0.6^+^
II1	Female	52	0.1	0.2	11	14	30.13	30.78	−15.00/−3.50*90 → 0.6	−17.50/−2.00*110 → 0.6
II2	Female	49	CF/30 cm	CF/10 cm	15	15	28.97	29.13	−18.50/−1.50*175 → 0.6^+^	−17.00/−1.50*180 → 0.03
II3	Female	47	0.2	0.3	15	13	30.43	30.11	−14.75/−0.75*50 → 0.6^+^	−15.00/−0.50*155 → 0.8

UCVA, Uncorrected Visual Acuity; IOP, Intraocular Pressure; AL, Axial Length; BCVA, Best Corrected Visual Acuity; OD, right eye; OS, left eye; /, missing examination data; CF, Counting Fingers; Ph, Pinhole visual acuity.

### 2.2 Sample collection

Participants should abstain from eating, drinking, or smoking for at least 30 min prior to sample collection. A sterile, disposable buccal swab was rubbed against the inner cheek mucosa 10 times, and this procedure was repeated on the opposite cheek to collect exfoliated oral mucosal cells. Immediately after collection, the swab was placed into a sample tube containing the preservative solution and vortexed 10 times to ensure thorough mixing. The sample was labeled with a unique identifier and transported to the Wenzhou Puxi Medical Laboratory at ambient temperature for subsequent sequencing.

### 2.3 Whole-exome sequencing and bioinformatics analysis

To conduct the analysis, DNA samples obtained from individuals affected by the condition underwent comprehensive sequencing of the entire exome. This process utilized the xGen Exome Research Panel V1.0 (Integrated DNA Technologies, San Diego, USA) and the NovaSeq 6000 platform (Illumina, San Diego, USA) for the sequencing procedure. The short-read sequence data were aligned to the hg19 human reference genome using the Burrows–Wheeler Aligner tool (BWA) (13) and variant calling with GATK (14) according to best practice guidelines. Variant annotation was conducted using ANNOVAR (15), and a series of in silico prediction tools were employed to evaluate the potential deleteriousness of missense and splice variants. These tools included Combined Annotation-Dependent Depletion (CADD) (16), REVEL (17), SpliceAI (18), SIFT (19), MutationTaster2021 (20), PolyPhen-2 (21), and AlphaMissense (22). The following thresholds were applied to define potentially deleterious variants: a CADD Phred-scaled score >15 (potentially deleterious), a REVEL score >0.5 (likely disease causing), a SpliceAI delta score >0.5 (potential splice-altering), a SIFT score <0.05 (damaging), and a PolyPhen-2 score >0.446 (deleterious). Variants predicted as “disease causing” by MutationTaster2021 or with an AlphaMissense score ≥0.564 (likely pathogenic) were also included. Variants meeting any of these criteria were flagged for further clinical evaluation. Prior to prioritization, all variants were filtered based on rarity (minor allele frequency <0.5%) and phenotype relevance. Given the rarity of causative coding variants in the Chinese population, only variants with a minor allele frequency below 0.5% were included. Population allele frequencies were referenced from gnomAD (23), ChinaMAP (24), and WBBC databases (25). Then disease and phenotype databases including Online Mendelian Inheritance in Man (OMIM),^1^ ClinVar,^2^ the Human Gene Mutation Database (HGMD),^3^ and Human Phenotype Ontology (HPO),^4^ were used for variant interpretation. The above database was accessed by June 2023. Variants that have been previously described to be disease-causing in the HGMD and literature were given the highest priority. The effects of mutations that were described in the HGMD were further validated by reading published literature reporting the variants, and only those variants that had convincing evidence were chosen. In addition, protein truncation mutations such as nonsense and frame shift (insertions or deletions) were also ranked higher in priority. Variants were categorized according to the 2015 guidelines of the American College of Medical Genetics and Genomics and the Association for Molecular Pathology (ACMG–AMP) (26). Variants were evaluated for the PP3 in-silico prediction (around 21 known insilico tools embedded in the database) criterion using predictions from Varsome.

### 2.4 Sanger sequencing validation

Sanger sequencing validation was performed by synthesizing primers targeting the DNA fragment of interest, followed by amplification by polymerase chain reaction (PCR). Sanger sequencing was then carried out using an ABI 3730xl sequencer (Applied Biosystems, USA). MutationMapper software was used to compare the obtained sequences with the reference sequence.

## 3 Results

### 3.1 Results of the initial phase

**Figure 2:**
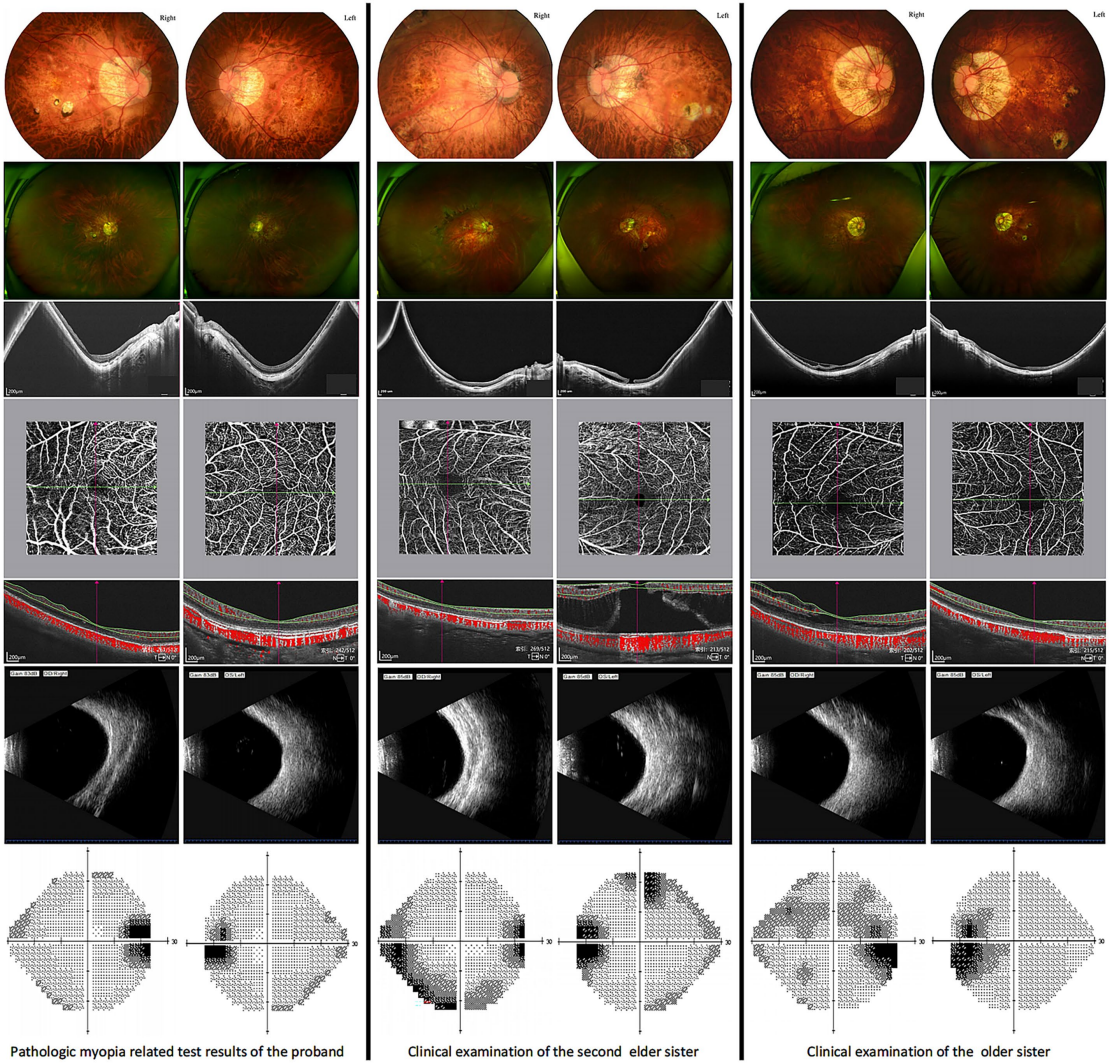
Ophthalmic examination for pathologic myopia of the proband and two sisters.

The fundamental clinical features of the family members in Figure 1 are summarized in Table 1. Examination Findings of Pathologic Myopia in Proband and Sisters, Refer to Figure 2 for detailed results.

#### 3.1.1 Genetic testing results

**Figure 3:**
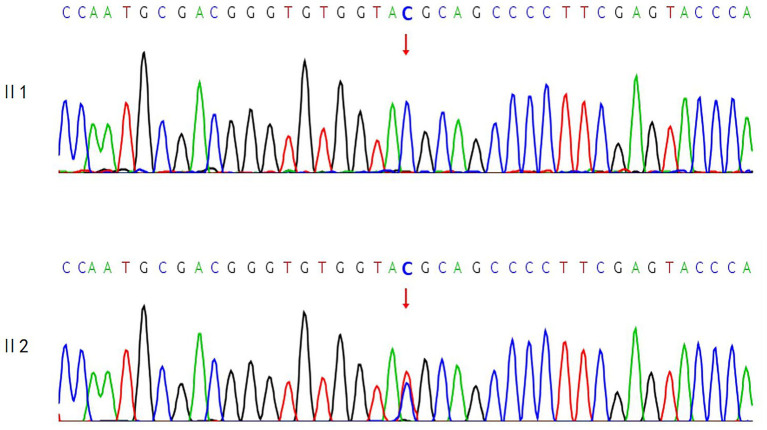
Sequencing chromatogram of the RHO gene in the proband’s eldest and second sisters.

A variant in the RHO gene was identified in the proband, which may be associated with the observed clinical phenotype: NM_000539.3:exon1:c.61C > T:p.R21C. Segregation analysis in the first generation revealed that the proband’s second sister was heterozygous for this variant, while the proband’s eldest sister exhibited the wild-type allele. The corresponding sequencing chromatogram is presented in Figure 3.

### 3.2 Results of stage II

**Figure 4:**
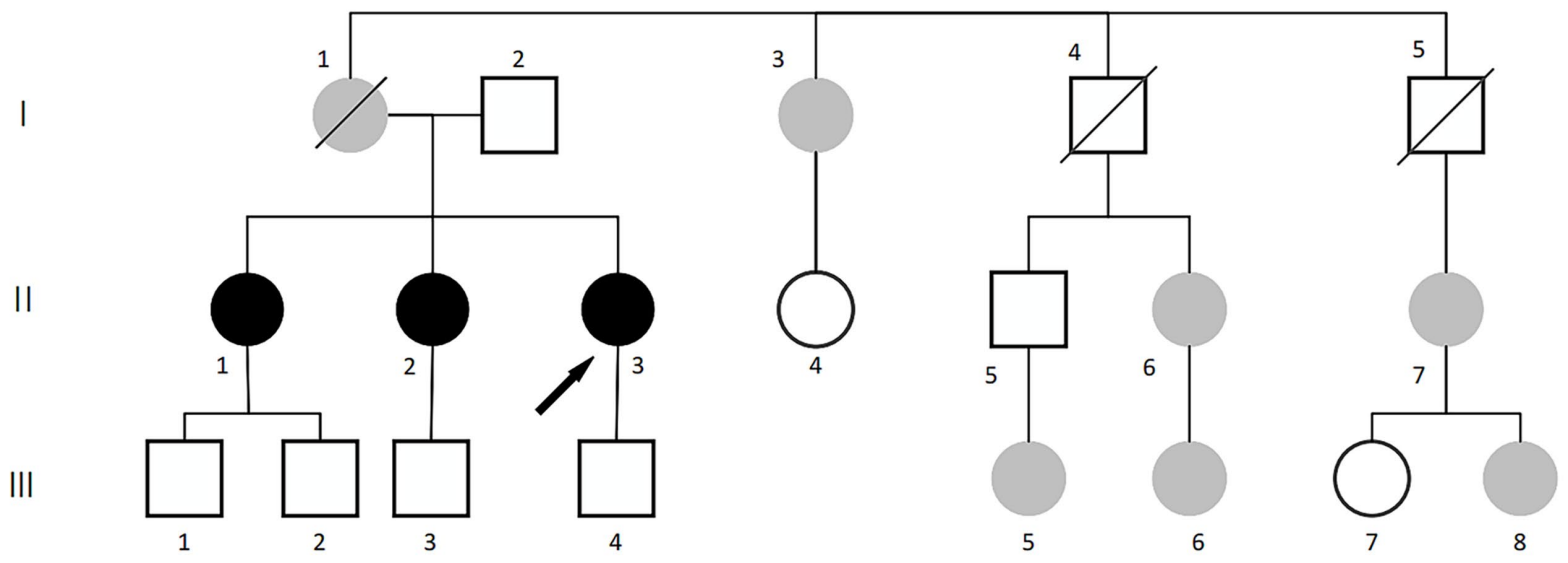
Pedigree of the family with extended testing for pathologic myopia. ●: females with pathologic myopia; 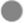: females with high myopia; 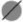: deceased females with high myopia; □: males without high myopia; ○: females without high myopia; 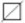: deceased males without high myopia; 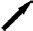: the proband.

A clustering of female-onset high myopia was observed among the mother’s siblings and their offspring. To enhance the detection power, we extended the screening for single-gene inherited ocular disorders to include maternal relatives with an axial length ≥ 26 mm and clinically significant high myopia: I3, II6 (right eye pathologic myopia and posterior scleral staphyloma), II7, III5, and III8. The family pedigree is shown in Figure 4. Given the passing of the proband’s mother, a key individual in the pedigree, we conducted additional testing on the father and the second sister, who had the most severe pathologic myopia among all family members, to investigate potential gene loci.

Table 2 summarizes the basic clinical characteristics of the remaining participants depicted in Figure 4.

**Table 2:** Ophthalmic examination parameters for additional members shown in Figure 4.

Member ID	Gender	Age(y)	UCVA		IOP(mmHg)		AL(mm)		Refraction & BCVA	
			OD	OS	OD	OS	OD	OS	OD	OS
Ι3	Female	57	0.05	0.2	15	14	26.44	23.81	−7.00/−1.75*110 → 0.6	−1.50/−1.25*70 → 0.9
Ι4	Male	/	/	/	/	/	/	/	/	/
Ι5	Male	/	/	/	/	/	/	/	/	/
II4	Female	32	0.2	0.2-	15	16	25.07	25.39	−4.00DS → 0.8-	−4.50DS → 1.0
II5	Male	47	1.2	1.2+	19	18	23.19	23.17	Not applicable	Not applicable
II6	Female	50	CF/10 cm	CF/30 cm	19	16	28.49	24.39	−15.00/−0.50*90 → 0.02	−3.00DS*80 → 0.12-
II7	Female	43	0.3	0.2	14	16	25.58	26.03	−7.25/−1.00*10 → 1.0-	−7.50/−2.25*175 → 1.0-
III1	Male	28	1.0	0.8	18	17	/	/	−2.00DS/−0.50 → 1.2	−2.00DS/−0.50 → 1.2
III2	Male	21	1.0	0.8+	17	14	23.67	23.91	Not applicable	Not applicable
III3	Male	27	0.15	0.7+	15	15	23.57	22.91	−2.50/−2.50*175 → 1.0-	0/−3.00*180 → 1.2
III4	Male	17	0.4	1.0	18	17	24.66	24.00	−1.25DS → 1.2	+0.50 → 1.2+
III5	Female	22	0.15	0.3	20	20	25.72	26.33	−6.00/−0.50 → 0.8	−7.50/−0.50 → 0.8
III6	Female	27	0.1	0.2	18	15	25.53	24.68	−6.00/−1.00*100 → 1.0	−3.25/−1.00*110 → 1.0+
III7	Female	23	0.15	0.2	14	13	23.53	23.43	−3.00/−0.50*180 → 1.0	−2.25/−0.50*175 → 1.0
III8	Female	12	0.1	0.1	20	19	26.10	25.74	−7.00/−3.50*175 → 0.8	−5.50/−4.00*180 → 0.8-

UCVA, Uncorrected Visual Acuity; IOP, Intraocular Pressure; AL, Axial Length; BCVA, Best Corrected Visual Acuity; OD, right eye; OS, left eye; /, missing examination data; CF, Counting Fingers; DS, Diopter Sphere; not applicable, myopia is nonexistent.

**Figure 5:**
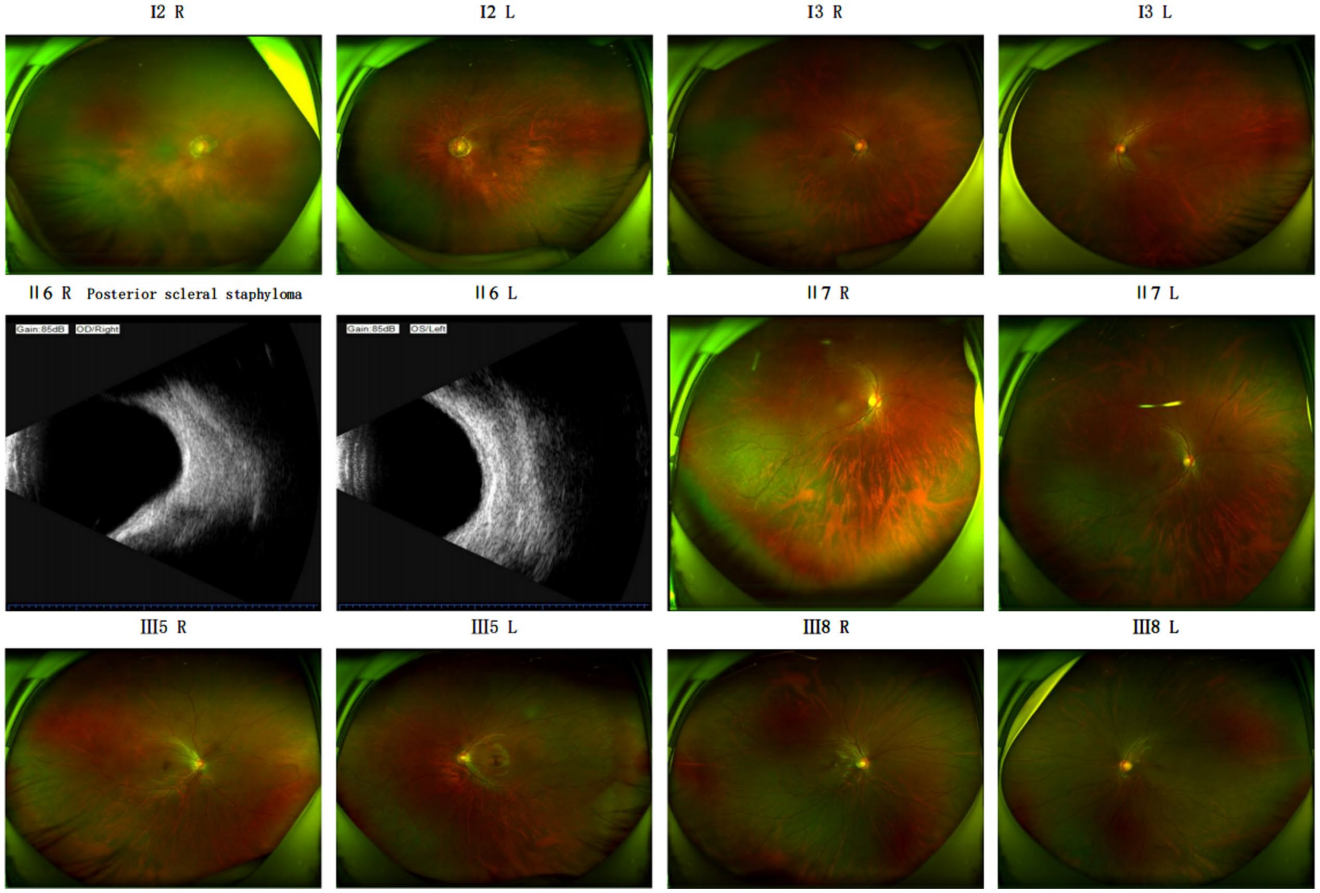
Funduscopic findings in extended family members (I3, II6, II7, III5, III8) and the proband’s father (I2).

Additional ophthalmic examinations, including fundus imaging and B-scan ultrasonography, were conducted for the extended family members (Ι3, II6, II7, III5, III8) and the proband’s father (I2) (Figure 5). The examination results for the second sister were consistent with those described previously.

Extended monogenic eye disease screening results for family members (Ι3, II6, II7, III5, III8), the proband’s father (Ι2), and the second sister (II2): Negative.

## 4 Discussion

In this study, we have identified a putative pathogenic variant in the RHO gene (NM_000539.3:exon1:c.61C > T:p.R21C) from a family with pathological myopia. The variant was classified as of uncertain clinical significance according to ACMG guidelines. This variant has been associated with several conditions: Congenital stationary night blindness, type 1A (CSNB1A) (MIM: 610445, inheritance: autosomal dominant [AD]); Retinitis pigmentosa, type 4 (RP4) (MIM: 613731, inheritance: [AD] or autosomal recessive [AR]); and Leber’s congenital amaurosis (LCA) (MIM: 136880, inheritance: [AD] or [AR]). This variant is located in exon 1 of the RHO gene, leading to a substitution of arginine at position 21 with cysteine (p.Arg21Cys). The RHO gene, located on chromosome 3q22.1, spans 1,047 base pairs and consists of five exons. It encodes the rhodopsin protein, which comprises 348 amino acids (27). Currently >150 different rhodopsin mutations have been identified, all contributing through multiple mechanisms with each having distinct consequences on the protein structure and function, leading to a wide range of clinical phenotypes (28). Variants in the RHO gene are a common cause of RP, less frequently associated with CSNB, and rarely with LCA (29, 30). The proband reported no history of night blindness; visual field index was 93% in the right eye and 89% in the left eye. No characteristic changes, such as osteocytic pigmentation, were observed in the fundus. Due to her lack of willingness to undergo further testing, full-field electroretinography could not be performed to investigate and diagnose CSNB or LCA. The proband and both of his sisters exhibited posterior scleral staphyloma and bilateral pathologic myopia, suggesting a strong familial aggregation of pathologic myopia within this family. The proband’s eldest sister was homozygous for the wild-type allele at the mutation site, while the second sister was heterozygous. As the proband’s mother had passed away, we were unable to obtain her sample, thereby limiting our ability to gather comprehensive genetic data. Consequently, whole-exome sequencing (WES) was conducted on the proband’s father and the second sister, who exhibited the most severe pathologic myopia-related complications, to identify potential disease-associated loci. However, no pathogenic variants were identified in either the father or the second sister that could explain the observed clinical phenotype. Based on these findings, it is hypothesized that the proband’s mother may have harbored germline mosaicism. Germline mosaicism involves the presence of two or more distinct genotypes in the germ cells, whereas a single genotype is typically present in somatic cells. This condition can lead to unpredictable transmission of genetic variations from mother to offspring, thereby explaining the non-Mendelian inheritance pattern observed in this family. A clustering of high myopia among female members was observed in the siblings and offspring of the proband’s maternal lineage. To enhance our ability to detect pathogenic variants, we expanded the scope of genetic screening for monogenic inherited eye diseases. We included individuals from the maternal family who exhibited an axial length greater than 26 mm and signs of high myopia: Ι3, II6 (right eye pathological myopia, right eye posterior scleral staphyloma), II7, III5, III8. Although WES did not identify any pathogenic variants associated with monogenic eye diseases in these individuals, the extension of the screening was aimed at detecting potential genetic variants that may have been overlooked in the proband and their immediate family. In the extended examination, individual II6 exhibited pathological myopia and staphyloma in the right eye, while the fundi of other individuals did not meet the diagnostic criteria for pathological myopia. However, a sample from individual Ι3 was the only one available for analysis among the first generation of the maternal lineage. Given that the fundus changes in individual II7 may progress to meet the diagnostic criteria for pathological myopia over time. Younger individuals III5 and III8 may develop progressive myopia and associated fundus changes as they age. Therefore, we decided to proceed with genetic testing for these individuals in the second phase to identify potential genetic risks at an early stage. This long-term follow-up and monitoring is crucial for understanding the natural history and genetic underpinnings of pathological myopia.

Although the RHO c.61C > T (p.R21C) variant is currently classified as a variant of uncertain significance (VUS), its occurrence in the proband with severe pathological myopia, along with the presence of the heterozygous state in her second sister with a similarly severe phenotype, suggests a potential, albeit not confirmed, role in disease expression. Given that RHO mutations have been traditionally associated with retinal dystrophies, this observation may point toward a broader phenotypic spectrum or gene–gene/environment interaction contributing to myopia pathogenesis. As such, reporting this variant adds to the known allelic and phenotypic diversity of RHO-related diseases and provides a valuable data point for future studies examining atypical presentations of RHO mutations in ocular disease.

Additionally, the proband, the mother, and the second sister all have hypothyroidism, whereas the father has hyperthyroidism, and the eldest sister exhibits normal thyroid function. These observations suggest that genetic factors may play a role in the thyroid dysfunction observed within this family. Previous studies have demonstrated that fundus alterations in pathological myopia are associated with increased levels of transthyroxin (TTR), whereas TTR levels are significantly reduced in the fundi of individuals with high myopia but without significant pathological changes (31). The functional impairment observed in the macular region of pathological myopia is associated with elevated TTR expression. Following the misfolding of TTR’s secondary structure, the protein loses its ability to bind to retinol-binding protein (RBP) (32). TTR is a homotetrameric protein found in serum and cerebrospinal fluid. As a carrier for thyroxine (T4) and retinol, TTR primarily facilitates the transport of thyroid hormones and also participates in the metabolic transport of vitamin A in conjunction with RBP. The RHO gene encodes rhodopsin, a light-sensitive receptor protein consisting of retinal and opsin, which plays a crucial role in phototransduction. While there may be complex interactions between thyroid dysfunction and pathological myopia, and TTR may serve as a molecular bridge between these conditions, this does not necessarily indicate a direct genetic link, particularly in relation to RHO gene mutations. To gain a more comprehensive understanding of this complex relationship, further targeted studies are required to elucidate the specific mechanisms involved.

Future research should focus on collecting additional DNA samples from extended family members, particularly younger individuals in the third generation, for longitudinal observation and potential identification of progressive changes. Moreover, functional studies—such as *in vitro* assays using retinal cell lines expressing the RHO p.R21C mutant protein—could help determine whether this substitution affects rhodopsin stability, trafficking, or phototransduction function. The use of CRISPR/Cas9-edited zebrafish or mouse models could also help establish any pathogenicity associated with this variant *in vivo*. These approaches would allow more definitive conclusions regarding the biological relevance of this variant and clarify its potential mechanistic link to high myopia.

The process of familial genetic testing for this disease has provided us with a deep understanding of the primary challenges in clinical family-based genetic testing. First, genetic heterogeneity complicates the identification of specific pathogenic variants. Pathological myopia can involve multiple genetic variations, necessitating the analysis of numerous candidate genes, which significantly increases both the cost and the duration of the testing process. Second, a significant number of detected variants are classified as VUS, including the RHO gene variant in the proband, which introduces uncertainty into clinical decision-making. Moreover, sample acquisition is challenging, and the processing requirements are stringent. Deceased or inaccessible family members (Ι1, Ι4, Ι5) restrict our ability to obtain comprehensive genetic data, thereby impacting the thorough understanding of inheritance patterns. Improper sample handling and storage can compromise the reliability of test results. Therefore, strict adherence to standard operating procedures (SOPs) during sample collection and processing is essential. The selection of appropriate technology and associated costs present additional significant challenges. Several genetic testing technologies are available, including Sanger sequencing, whole-exome sequencing, whole-genome sequencing, and others, each with distinct advantages and limitations. Selecting the most suitable detection technology necessitates a comprehensive evaluation of the testing objectives, budget constraints, and laboratory capabilities. While high-throughput sequencing technology has enhanced detection efficiency, its high cost remains a significant financial burden for many medical institutions and individual patients, particularly in family studies requiring multiple tests or involving multiple family members, where the economic pressure is especially pronounced. The rapid advancement of genetic testing and genetics encompasses multidisciplinary knowledge. Continuous learning and updating of this knowledge are essential, and collaboration among clinicians, researchers, geneticists, and molecular biologists is crucial for enhancing the interpretation and application of test results.

## 5 Conclusion

During the search for pathogenic genes, this family faced several challenges, including difficulties in sample acquisition, the selection of appropriate sequencing technologies, and genetic heterogeneity, all of which increased the complexity of identifying specific pathogenic variants and influenced clinical decision-making. Nevertheless, this study offers valuable insights into the genetic underpinnings of pathological myopia and underscores the importance of further research, particularly in exploring polygenic inheritance patterns and gonadal mosaicism. The clinical significance of the RHO gene variant NM_000539.3:exon1:c.61C > T:p.R21C in this family with pathological myopia remains unclear, although this variant may be associated with the clinical phenotype of pathological myopia and its associated complications. Given the inconsistent genotype–phenotype correlations observed among family members, it is hypothesized that the proband’s mother may harbor a gonadal chimeric variant, which could account for the variability in disease presentation within the family. Future research will require additional samples and in-depth genetic analyses, particularly focusing on gonadal mosaicism, to comprehensively elucidate the polygenic inheritance pattern and underlying mechanisms of pathological myopia. In conclusion, although the RHO c.61C > T:p.R21C variant identified in this family has not yet demonstrated a definitive genotype–phenotype correlation, its potential involvement in familial pathologic myopia cannot be entirely excluded. The unusual inheritance pattern and clinical heterogeneity, particularly in the maternal lineage, raise the possibility of germline mosaicism or polygenic effects. This case emphasizes the complexity of interpreting rare genetic variants in familial myopia and underscores the importance of integrating clinical, genetic, and functional data. Further studies with expanded sample sizes and functional validation are warranted to determine whether this variant represents a true modifier or driver of disease phenotype.

## Data Availability

The original contributions presented in the study are included in the article/supplementary material, further inquiries can be directed to the corresponding author.

## Footnotes

^1^ http://www.omim.org

^2^ http://www.ncbi.nlm.nih.gov/clinvar

^3^ http://www.hgmd.org

^4^ https://hpo.jax.org/app/
